# Eph A10-modified pH-sensitive liposomes loaded with novel triphenylphosphine–docetaxel conjugate possess hierarchical targetability and sufficient antitumor effect both *in vitro* and *in vivo*

**DOI:** 10.1080/10717544.2018.1446475

**Published:** 2018-03-07

**Authors:** Jiulong Zhang, Chunrong Yang, Shuang Pan, Menghao Shi, Jie Li, Haiyang Hu, Mingxi Qiao, Dawei Chen, Xiuli Zhao

**Affiliations:** a School of Pharmacy, Shenyang Pharmaceutical University, Shenyang, Liaoning, PR China;; b College Pharmacy, Jiamusi University, Jiamusi, Heilongjiang, PR China;; c Mudanjiang Medical University, Mudanjiang, Heilongjiang, PR China

**Keywords:** pH-responsive, Docetaxel conjugate, multifunctional liposomes, hierarchical targetability, anticancer efficacy

## Abstract

Mitochondrial-targeting therapy was considered to be a promising approach for the efficient treatment of cancer while positive charge induced nonspecific cytotoxicity severely limits its application. To overcome this drawback, a novel mitochondria targeted conjugate triphenylphosphine-docetaxel (TD) has been synthesized successfully and incorporated it into liposomes (EPSLP/TD), which possessed excellent pH-sensitive characteristic, EphA 10 mediated active targetability as well as mitochondria-targeting capability. EPSLP/TD was characterized to have a small particle size, high-encapsulation efficiency and excellent pH-sensitive characteristic. Compared with DTX-loaded liposomes (EPSLP/DTX), EPSLP/TD possessed higher cytotoxicity against MCF-7 cell line. Mitochondrial-targeting assay demonstrated mitochondria-targeting moiety triphenylphosphine (TPP) could efficiently deliver DTX to mitochondria. Western immunoblotting assay indicated that EPSLP/TD could efficiently deliver antitumor drug to mitochondria and induce cell apoptosis via mitochondria-mediated apoptosis pathway. *In vivo* antitumor study demonstrated EPSLP/TD owed excellent *in vivo* antitumor activity. Histological assay demonstrated EPSLP/TD showed strongly apoptosis inducing effect, anti-proliferation effect and anti-angiogenesis effect. This work investigated the potential of hierarchical targeting pH-sensitive liposomes is a suitable carrier to activate mitochondria-mediated apoptosis pathway for cancer therapy.

## 1. Introduction

Cancer is a great challenge to human’s health over last few decades (Malhi et al., 2012; Noh et al., 2015; Pérez-Herrero and Fernández-Medarde, 2015; Yang et al., 2015; Abdel Aziz et al., 2016). Among all of the treatment against cancer, chemotherapy is considered to be one of the most widely used application in clinical (Wang et al., 2011; Li et al., 2013a; Ma et al., 2013; Zhou et al., 2013). However, severe systematic toxicity hindered its further application. Therefore, reducing side effects, times of administration as well as increasing therapeutical effect are becoming a hot plot of research in drug delivery system (DDS) (Wong et al., 2013; Gaowa et al., 2014). Target drug delivery offers a potential strategy for the chemotherapy.

In general, antitumor drugs kill tumor cells by two basic approaches. One is to kill cancer cells by directly exposing them to toxic chemicals. Another is to induce the suicide of cancer cells apoptosis (Zhou et al., 2013). There are two major apoptosis pathways: extrinsic and intrinsic pathways, which both activate the caspases. The extrinsic is triggered at plasma membrane through activating the death receptors. The intrinsic pathway is triggered by various apoptotic stress signals and is characterized by mitochondrial dysfunction and activation of caspases-9 and caspases-3. Both of them take place in mitochondria of cancer cells (Yang et al., 2014). Therefore, mitochondria was considered to be an important target site for the delivering of antitumor drugs. Docetaxel (DTX), a microtubule stabilizer, which binds to the N-terminal region of the β-tubulin and stabilize microtubules, thereby preventing the activation of tumor cell division and cell cycle, is widely used as a chemotherapeutic drug such as breast, ovarian, nonsmall cell lung, prostate and gastric cancers (Baati et al., 2015; Inada et al., 2015; Ma et al., 2016). Over the last few decades, increasing evidences suggested that DTX could activate apoptosis by reducing the mitochondrial membrane potential and increasing the permeability of mitochondria, which resulting in the activation of cell apoptosis (Huang et al., 2014; Chatterjee et al., 2015; Lin et al., 2015).

Due to the key role in apoptosis, mitochondria was considered to be a key target to ‘cutoff’ intracellular energy supply and induce cell apoptosis by preparing mitochondrial-targeting liposomes to deliver antitumor drugs. In general, mitochondria membrane potential kept negative in normal environment. Therefore, electrostatic interaction is a possible strategy for mitochondria target. There are many ligands which have positive charge, for example: triphenylphosphine (TPP) (Li et al., 2015a), dequalinium (DQA) (Tang et al., 2005; García-Pérez et al., 2014) and mitochondria targeting signal peptides (MTSs) (Shani et al., 2011; Kawamura et al., 2013), etc. However, when the moiety was modified on the surface of liposomes, positive surface charge could be found and nonspecific toxicity is becoming an important problem (Torchilin, 2014).

Liposomes are able to enhance pharmaceutical properties and decrease systematically toxicity. In general, liposomes were consisting of phosphatidylcholine (PC) and cholesterol. Due to its particle size distribution (PSD) is approximately 100 nm, liposomes could specifically accumulate into the tumor sites *via* enhance permeability and retention effect (EPR). Polyethylene glycol (PEG) was used to modify on the surface of liposomes to reach long circulation and avoid elimination by the reticuloendothelial system (RES) in blood system (Şen Karaman et al., 2014; Zhang et al., 2014; Mabuchi et al., 2015). However, a major barrier of this method is PEG shell could shield drug release, which resulting in insufficient therapeutical effect even though the preparation is reached to the tumor sites (Hatakeyama et al., 2011; Ai et al., 2014; Cui et al., 2015).

It is well known that the pH of tumor microenvironment is lower than normal tissue, which is 6.0 to 5.0 in early endosomes and late lysosomes, respectively (Yu *et al.*, 2014; Du et al., 2015; Choi et al., 2015). How to avoid degradation of lysosomes and release drugs into cytoplasm are great challenges for intracellular drug delivery. Dioleoylphosphoethanolamine (DOPE), a well-known biomaterial which has pH-sensitive characteristic, is suitable for the preparation of liposomes, which alone form a hexagonal phase (H_II_), into a lamellar vesicle at physiological pH while inducing content leakage at acidic pH (Osanai and Nakamura, 2000). After initialization, DOPE could merge with lysosome membrane in tumor microenvironment (lysosome pH) and release drugs to cytosol (Romberg et al., 2008; Li et al., 2013b; Bowey et al., 2014). However, the major problem of this method is that when the lysosome membrane becomes instable, it is the free drug but not the preparation that release from lysosomes, which resulting in insufficient mitochondrial-targeting capability.

To overcome these drawbacks, in this paper, we synthesized TPP-DTX (shorten as TD) as a mitochondrial-targeting antitumor drug. Our group has synthesized a PEG-Schiff base-cholesterol (PSC) derivate which showed significant pH-sensitive feature and used it to modify on the surface of the liposomes (PSLP) (Chen et al., 2016). Although liposomes could accumulate in tumor site *via* EPR-mediated passive targetability, the drug concentration in cytoplasm is limited. It has been reported that receptor mediated cell endocytosis with modification of specific ligands could significantly increase the drug concentration into cytoplasm (Qiu et al., 2016a). Therefore, we are interested to use this strategy to reach higher therapeutical activity. Eph receptor A10 (EphA 10) has been reported to be an ephrin receptor family protein, which involved in cancer progression. Furthermore, EphA10 receptor has been widely expressed in breast cancer (MCF-7), while this receptor was hardly expressed in normal tissue (except testis) (Aasheim et al., 2005; Zang et al., 2016). This characteristic could significantly decrease the nonspecific toxicity and reach higher therapeutical effect. Therefore, EphA10 is a suitable targeting site for breast cancer therapy. In general, most of DDS was designed to target the surface of tumor cells. However, efficient functional organelle delivery of therapeutical agents has not been extensively investigated. It is well known that most of antitumor drugs could be utilized only in specific organelles. Therefore, specific organelle is also an important targeting site which should not be neglected, while it is necessary to design novel DDS which could not only target tumor cells but also target specific organelles of tumor cells. This strategy is usually called ‘hierarchical target’. In this paper, EphA10 antibody was utilized to modify on the surface of pH-sensitive liposomes (EPSLP) and mitochondrial targeting derivate TD was incorporated into the liposomes (EPSLP/TD). When this preparation was intravenous administration in blood system, PEG shell of liposomes could prevent reorganization of RES and stay stable. These liposomes could accumulate in tumor tissue *via* EPR effect and be internalized into cytoplasm *via* receptor-mediated cell endocytosis. In the acidic microenvironment of tumor cells, Schiff base bond would hydrolysis due to its pH-sensitive feature and PEG shell would remove from the liposomes. Drug could release into cytoplasm and accumulate into mitochondria which possessed hierarchical targetability, followed by inducing cell apoptosis to reach higher antitumor activity. Particle size, drug loading content, *in vitro* drug release behavior of liposomes were investigated. Cell cytotoxicity, cellular uptake and apoptosis inducing effect of different liposomal formulations were demonstrated as well. A series of trials suggested EPSLP/TD could induce mitochondria-mediated cell apoptosis. *In vivo* antitumor activity was demonstrated to evaluate its therapeutical effect. Furthermore, we scarified the tumor tissues and evaluate its antitumor efficacy in histological level. This method supported favorable evidences for therapeutical effect.

## 2. Methods and materials

### 2.1. Materials

(4-Carboxybutyl) triphenylphosphonium bromide (TPP-COOH) was obtained from Aladdin (Shanghai, China). Docetaxel (DTX) was supplied from Melone Biotechnology Co. Ltd (Dalian, China), 1-(3-dimethylaminopropyl)-3-ethylcarbodiimide hydrochloride (EDC), 4-dimethylaminopyridine (DMAP) were both obtained from Shanghai Medpep Co. Ltd, China. Fluorescein isothiocyanate (FITC) was supplied from Sigma Aldrich (Germany). Soybean phospholipid (SPC), cholesterol (CH), dioleoylphosphoethanolamine (DOPE) were all purchased from A.V.T. Pharmaceutical Science and Technology Co., Ltd (Shanghai, China). N, N-disuccinimidyl carbonate (DSC) from Shanghai Chemical Reagent Co. Ltd. (Shanghai, China). EphA 10 antibody (ab106437) was purchased from Abcam (England). 5-diphenyltetrazolium bromide (MTT) was purchased from Sigma Aldrich (Germany). Dulbecco’s modified Eagle medium (DMEM), RPMI 1640 medium and fetal bovine serum (FBS) were purchased from Gibco BRL (MD, USA). Annexin V-FITC/PI cell apoptosis detection kit, mitochondrial membrane potential detection kit were both supplied from Beyotime Biological Technology Co. Ltd (Shanghai, China) MitoTracker Red was provided from Invitrogen (USA). Bax, Bcl-2, caspases-3 and caspases-9 primary antibody were purchased from R&D (USA). PARP, cleaved-PARP and Ki-67 primary antibody were purchased from Proteintech group (USA). Cytochrome-C primary antibody was purchased from Abcam (Germany). Cy3-Labeled Goat Anti-Mouse IgG was purchased from Beyotime biotechnology (China). Biotin-streptavidin HRP detection Kit, DAB Kit were purchased from Zhongshan Goldenbridge biotechnology (Beijing, China). Ultrasensitive streptavidin peroxidase immunohistochemistry Kit was purchased from Maixin biotechnology (Jiangsu, China). *In situ* cell death detection Kit, POD was purchased from Roche. All other reagents were analytical grade.

### 2.2. Synthesis of TD

TPP-COOH (0.25 mmol), DTX (0.25 mmol), EDC (0.5 mmol) and DMAP (0.5 mmol) were dissolved in anhydrous dimethyl sulfoxide (DMSO). The mixture was stirred at room temperature for 48 h under the protection of nitrogen. Then, the mixture was transformed into dialysis bag (MWCO: 2.0 kDa) and dialyzed against distilled water to remove excess EDC and DMAP. Precipitation was collected and extracted using dichloromethane (DCM). The raw product was purified through silica gel column. The final product (TD) was characterized and identified by nuclear magnetic resonance spectroscopy (400 MHz ^1^H NMR, Bruker AVANCE III) and matrix assisted laser desorption ionization time of flight mass spectrometer (MALDI-TOF MS).

### 2.3. Synthesis of TPP-FITC

FITC (0.04 mmol), TPP-COOH (0.04 mmol), EDC (0.1 mmol) and DMAP (0.1 mmol) were dissolved in 5 mL of anhydrous DMSO. The mixture was allowed to react for 48 h under protection of nitrogen. Then, the mixture was transferred into a dialysis bag (MWCO: 1.0 KDa) and dialyzed against deionized water for 3 days. The final solution was lyophilized and TPP-FITC (shorten as TF) was received. TF was characterized and identified by ^1^H NMR and MALDI-TOF MS.

### 2.4. *In vitro* stability of TD

High-performance liquid chromatography (HPLC) was used to evaluate the stability of TD in acidic tumor microenvironment. In brief, 2 mg of TD was dissolved in 10 mL PBS solution containing 0.3% Tween 80 with different pH (pH 7.4 and 5.0). The sample was shaken in shaking incubator with the stirring speed of 100 rpm at 37 °C. At 0, 2, 4, 6 h, 1 mL sample was taken and filtered through 0.22-μm membrane. The sample was finally detected.

### 2.5. Preparation of EphA10-targeted liposomes

The synthesis procedure of pH-sensitive PSC has been reported in our previous study (Chen et al., 2016). The end group –NH_2_ could be used to further modify to reach active targetability. For the modification of EphA10, PSC-NH-maleimide was synthesized at the beginning of the preparation according to the reference with minor modification (Li et al., 2015b). In brief, PSC and DSC were dissolved in N, N-dimethylformamide (DMF) at a molar ratio of 1:1 and the mixture was stirred at room temperature for 4 h. This solution was dialyzed against distilled water for 2 days with a dialysis membrane (MWCO 1.0 kDa) and then lyophilized to obtain PSC-NH-maleimide (PSC-Mal). Nontargeted liposomes (PSLP/DTX or PSLP/TD) were prepared by a thin-film hydration method. In brief, DTX or TD: SPC: DOPE: Chol: PSC-Mal = 4: 40: 40: 5: 5 (w/w, total lipid content: 20 mg mL^−1^) were dissolved in chloroform, the organic solvent was removed using rotary evaporator at 40 °C. The thin film was hydrated using distilled water at 50 °C for 1 h and 2 min bath-type sonication. Liposomes were further sonicated with a probe-type sonicator at 300 W power (carried out every 1 s for a 3 s duration in ice bath) for 3 min. Finally, the liposomes was filtered through 0.22-μm filter.

For the preparation of EphA 10-modified liposomes (EPSLP/DTX or EPSLP/TD), EphA 10 was incubated with non-targeted liposomes overnight at 4 °C at the initial ratio of 100 μg EphA10 per 500 μL liposomes. Unreacted EphA 10 was removed using a Sepharose CL-4B column eluted with buffer (10 mmol L^−1^ Na_2_HPO_4_, 2 mmol L^−1^ KH_2_PO_4_, 136.8 mmol L^−1^ NaCl, pH 7.4).

In order to further demonstrate whether the pH-sensitivity of PSC was resulted in the cleavage of Schiff base bond. We used Mal-PEG_2000_-Cholesterol derivate (Shanghai Ponsure Biotechnology, China) to replace PSC-Mal as control to prepare pH-nonsensitive liposomes (EPELP/TD). LP/DTX or LP/TD were prepared using the same way except PSC was replaced by cholesterol. Different FITC or TF-loaded liposomes were prepared with the same method as previously described.

### 2.6. Characterization of different liposomes

The encapsulation efficiency (EE %) and loading content (LC %) of different liposomes were measured using Sephadex G50 mini-column centrifugation method. The amount of DTX or TD was calculated from the calibration curve and the linear range was between 1-20 μg mL ^1^ using high performance liquid column (HPLC) method with the detection wavelength of 230 nm.

The encapsulation efficiency (EE %) of liposomes was calculated using the equation:

$$\text{EE\%}=\frac{W}{W_{0}}\times 100$$

where *W* was the amount of TD or DTX in liposomes after filtration. *W_0_* indicated the concentration of TD initially added in liposome preparation.

The loading content (LC %) of liposomes was calculated using this formula:

$$\text{LC \%}=\;\frac{W_{\text{drug}}}{W_{\text{total}}\;}\times 100$$

where *W*_drug_ was the weight of the drug and *W*_total_ was the weight of all the components.

The particle size and zeta potential of liposomes were measured using a Dynamic Light Scattering (DLS) Analyzer (Malvern ZetasizerNano ZS90). The morphology of liposomes was observed using JEM-100CX transmission electron microscope (TEM).

### 2.7. *In vitro* drug release of liposomes

The release profiles of DTX or TD from different liposomal formulations were investigated using a dialysis bag (MWCO 3.5KDa) at 37 °C. In brief, appropriate volume (contained 2.0 mg of TD or DTX) of liposomes were transferred into a dialysis bag and placed in a conical flask with 100 mL PBS solution (at pH 7.4 and 5.0) which contained 0.5% Tween 80 to meet sink condition. All the flasks were shaken in shaking incubator with the stirring speed of 100 rpm. At different time point, 1.0 mL of samples were taken and equal volume of blank PBS solution was added to maintain the total volume. Concentration of DTX or TD were measured using high-performance liquid chromatography (HPLC) in a following conditions: Diamonsil C18 column (250 × 4.6 mm i.d., pore size 5 μm); the mobile phase was acetonitrile: water (50:50, v/v); flow rate, 1.0 mL min^−1^; measuring wavelength 230 nm (Ai et al., 2014). The cumulative percentage drug release (Er) was calculated using this formula:

$$Er=\frac{V_{0}\sum_{i=1}^{n-1}C_{i}+V_{0}C_{m}}{{\text{m}}_{\text{drug}}}\times 100\%$$

where m_drug_ represents the amount of TD or DTX in the liposomes, *V_0_* represents the total volume of the release medium. *C_i_* and *C_m_*represent the total concentration of TD or DTX in dialysis bag and concentration of TD or DTX in the *i* th sample, respectively.

### 2.8. Cell culture

Human breast cancer cells (MCF-7), human liver hepatocellular carcinoma (HepG2) and human lung adenocarcinoma cells (A549) cells were all purchased from Chinese Academy of Sciences (Shanghai, China). Three different cell lines were all grown in Dulbecco’s modified eagle medium (DMEM, Gibco, USA) supplemented with 10% fetal bovine serum (FBS) and 1% antibiotics (penicillin 100 U mL^−1^, and streptomycin 100 mg mL^−1^). Cells were grown under the condition of 5% CO_2_ at 37 °C.

### 2.9. *In vitro* cell cytotoxicity

Cell cytotoxicity of blank liposomes was measured using standard MTT assay. In brief, cells were seeded in 96-well plate (1 × 10^4^ cells per well). After culturing for 24 h, cells were then incubated with different blank liposomes or different drug-loaded formulations in a concentration gradient at 37 °C. After 24 h, 10 μL of MTT (5 mg mL^−1^) was added in each well and then further incubated for 6 h. Medium of each well was removed and 100 μL of DMSO was added to dissolve the internalized purple formazan crystals, followed by recording the absorbance at 490 nm using a BioRed microplate reader (MK3, Thermo, USA). The cell viability (%) was calculated using the following formula:

$$\text{Cell viability}\left(\%\right)=\frac{A_{\text{s}\text{a}\text{m}\text{p}\text{l}\text{e}}-A_{\text{b}\text{l}\text{a}\text{n}\text{k}}}{A_{\text{c}\text{o}\text{n}\text{t}\text{r}\text{o}\text{l}}-A_{\text{b}\text{l}\text{a}\text{n}\text{k}}}\times 100$$

where *A*_control_ and *A*_sample_ are the absorbance in the absence and in the presence of sample treatment, respectively. *A*_blank_ is in the absorbance of medium.

### 2.10. Cellular uptake assay

Cellular uptake of different fluorescence probe-loaded liposomes was measured using fluorescence microscopy. MCF-7 cells were seeded in 6-well plate at a density of 5 × 10^5^ cells per well and incubated overnight for attachment. Serum-free 1640 medium was added into each well and different formulations were added to reach the final concentration of probe was 20 nmol mL^−1^. Control group was performed by adding equal volume blank medium. After incubation for different time, the cells were washed using cold PBS solution for twice and fixed with 4% paraformaldehyde. The cells were subsequently stained with Hoechst 33258 (10 μgmL^−1^) and observed by fluorescence microscopy.

In order to quantitatively analyze the amount of cellular uptake of different formulations, flow cytometry was carried out. In brief, MCF-7 cells was seeded in 6-well plate at a density of 4 × 10^5^ per well and incubated overnight for attachment. Different formulations were allowed to add in each well and incubate for different time with the final concentration of fluorescence probe was 50 nmol mL^−1^. The cells were washed using PBS for twice, harvested and resuspended in 0.5 mL PBS for flow cytometry analysis. All the data were analyzed in triplicate.

### 2.11. Apoptosis-inducing effect study

Cell apoptosis experiment was measured using PI Annexin V-FITC dual-staining kit. The procedure was carried out in accordance with the guide of the kit. Briefly, cells were seeded in 6-well plate at a density of 5 × 10^5^ cells per well. Serum-free RPMI 1640 culture medium was added into each well. Then different formulations were added to each well to reach the final concentration of drug was 5 nmol mL^−1^. The cells were allowed to incubate with preparations for 24 h. Control group was performed by adding culture medium. Then, the cells were possessed according to the protocol of the kit and analyzed using flow cytometry. Each assay was repeated in triplicate.

### 2.12. Mitochondrial targeting assay

#### 2.12.1. Colocalization into mitochondria

Confocal laser scanning fluorescent microscopy (CLSM) was utilized to observe the co-localization between mitochondria and different formulations. Briefly, MCF-7 cells were seeded in a glass bottom dished at a density of 1 × 10^5^ cells per well and cultured overnight for attachment. Then the medium was replaced with serum-free RPMI 1640 and different formulations were added into each well for 4 h. The final concentration of the TF or FITC was 20 nmol mL^−1^. After incubation, cells were washed using PBS for twice and stained with Mitotracker Red for 30 min under the condition of 5% CO_2_ at 37 °C. The cells were washed twice using PBS, fixed with 4% paraformaldehyde and observed using CLSM.

#### 2.12.2. Drug content in the isolated mitochondrial fraction

Drug accumulation in mitochondrial was quantified by flow cytometry. In brief, cells were seeded in 6-well plate (1 × 10^5^ cells per well) and then different FITC or TF-loaded formulations were added to each well to reach the final concentration was 20 nmol mL^−1^. Control experiment was performed by adding culture medium. After incubation for 4 h, the cells were washed using PBS solution for twice. Mitochondrial isolation was carried out using a cell mitochondria isolation Kit. Mitochondria uptake of different formulations was measured using flow cytometry. Each sample was repeated in triplicate.

#### 2.12.3. Mitochondrial membrane potential (△ψm)

Mitochondrial membrane potential was measured using JC-1 fluorescence dye and analyzed using flow cytometry. In brief, cells were seeded in 6-well plate (1 × 10^6^ cells per well) overnight. Then, different formulations were added to each well and incubated for 12 h with the final concentration of DTX or TD was 5 nmol mL^−1^. The cells were washed using PBS solution for twice and incubated with JC-1 (5 μg mL^−1^) at 37 °C for 20 min in the dark. Then, the cells were washed using PBS for twice and analyzed by flow cytometry. Each sample was replicated for triplicate.

#### 2.12.4. Release of cytochrome c

Cytochrome *c* release from mitochondria of cells was measured using standard immunofluorescence assay. In brief, MCF-7 cells were seeded into 6-well plate (one coverslip in each well) at a density of 1 × 10^5^ cells per well. Different DTX or TD-loaded formulations were added into each well with the final concentration of drug was 5 nmol mL^−1^ and incubated for 24 h. Control group was performed by adding culture medium. Then, cells were fixed using cold acetone for 15 min, then washed with PBS and the following operations were according to the manufacture of the immunofluorescence assay.

### 2.13. Western immunoblotting assay

The expression of related protein was measured by western immunoblotting. In brief, cells were seeded in 6 plates well at a density of 1 × 10^6^ cells per well and incubated overnight to allow attachment. Different formulations were added into each well to make the final concentration of drug was 5 nmol mL^−1^ and further incubated for 24 h. Then, the cells were washed using cold PBS for twice and solubilized with lysis buffer (1% SDS, 100 mmol L^−1^ sodium fluoride, 50 mmol L^−1^ HEPES, 1% Triton X-100, 1 mmol L^−1^ phenylmethylsulfonylfluoride, 1 mmol L^−1^ EDTA, 2 mmol L^−1^ leupeptin and 1 mmol L^−1^ aprotinin). The samples were incubated in ice-bath for 30 min and vortex for 5 min, followed by centrifuging for 10 min at 12000 rpm and supernatant was collected to determinate the protein concentration using BCA Protein Assay Kit. The supernatant were separated by 12% SDS-PAGE and transferred to PVDF membranes. The membrane was blocked using 5% nonfat milk powder in PBS with Tween-80 (PBST) for 1 h, primary antibody was added and incubated overnight at 4 °C. The membrane was washed and incubated with secondary antibody. Labels were detected with enhanced chemiluminescence plus (ECL-plus) and autoradiography.

### 2.14. Animal tumor xenograft models

Female BALB/c nude mice were purchased from Shenyang Changsheng Biotechnology Co. Ltd. All the mice with subcutaneous tumors were established by injecting MCF-7 cells (approximately 1 × 10^6^ cells, suspended in 200 μL PBS) into the right backs of mice under anesthesia according to the reference (Zheng et al., 2012). When the tumor volumes reached 100–500 mm^3^, the *in vivo* trial was carried out. All the animal experiments were performed in accordance with the Experimental Animal Administrative Committee of Shenyang Pharmaceutical University

#### 2.14.1. *In vivo* biodistribution assay

In order to evaluate the targetability of EPSLP, *in vivo* biodistribution assay was carried out in nude mice bearing MCF-7 tumor cells (tumor volume was appropriately 500 mm^3^). 1, 1′-dioctadecyl-3, 3, 3′, 3′- tetramethyl indotricarbocyanine iodide (DiR, Invitrogen, USA) was invoked as a fluorescence probe and incorporated it into different liposomes. Preparation of different DiR-liposomes was in the same way as EPSLP/TD. Nude mice bearing MCF-7 tumor cells were injected intravenously *via* tail vein with the same DiR concentration. At different time point, the mice were anesthetized and imaged using *in vivo* image system (Carestream, USA).

#### 2.14.2. *In vivo* antitumor activity

In this study, nude mice-bearing MCF-7 tumor cells (tumor volume was appropriately 100 mm^3^) were carried out and the mice were sorted randomly to several groups (*n* = 6). Each group was injected different formulations through tail vein every 2 days for 4 times. Body weight and tumor volume were measured every 2 days. Tumor volume (mm^3^) was calculated using the formula: V = 1/2 × LW^2^ (L is the long diameter and W is the short diameter of the tumor tissue). At the 16th day, tumor tissues were harvested and weighted. At the end of trial, tumor tissues were performed and fixed using formalin for further assay.

### 2.15. Histological assay

In order to evaluate the influence of different formulations against tumor tissue, various studies were used in this section. Formalin-fixed tumor tissues were processed conventionally for paraffin-embedded tumor slides (5 μm thick). In order to investigate the anti-proliferative effect and anti-angiogenesis effect, Ki67 and CD31 immunohistochemistry was carried out assessed with anti-Ki67 antibody and anti-CD31 antibody, respectively. Ki67 and CD31 index was calculated as the ratio of proliferative cells to total cells in each field, using several random fields. TUNEL assays were used to evaluate the *in situ* apoptosis effect of tumor tissues using *in situ* cell death detection kit-POD. All the operations were according the instruction supplied by the kit.

### 2.16. *In vivo* toxicity

Healthy SCID mice were randomly divided into several groups (*n* = 6) and different formulations were injected through tail vein every 2 days. Body weight of the mice was measured every day and the behaviors were assessed. After 8 days postdosing, the mice were allowed to recover and they were weighted for another 6 days. At last, main organs were sacrificed and washed using PBS, fixed in 4% formaldehyde, embedded in paraffin and cut for H&E staining.

### 2.17. Statistical analysis

The data were reported as means ± standard division (SD). Statistical significance was investigated using student’s *t*-test or one-way ANOVA. *p* < .05 was considered to be significant difference. All data were measured in triplicate.

## 3. Results and discussions

### 3.1. Synthesis and characterization of TD and TF

The synthesis route of TD has been shown in Figure S1 (A). In brief, carboxyl group of TPP-COOH was reacted with hydroxyl group of DTX *via* simple ester reaction. The raw product was purified using silica gel column. The product was characterized using ^1^H-NMR. As was shown in Figure S1 (B), it illustrates the ^1^H-NMR spectra of DTX and TD, respectively. The signals of protons from TPP-COOH were at 7.70–8.00 ppm and there is no peak in the ^1^H-NMR of DTX. However, this signal appeared in the ^1^H-NMR spectra of TD. Furthermore, MALDI-TOF MS (Figure S1 (C)) was used to detect the exact mass of TD and the strongest intensity peak (*m*/*z* = 1152.50126) was assigned as [M + H] ^+^ of TD, indicating that TPP was successfully conjugated to DTX.

In order to determinate whether this conjugate could accumulate in mitochondria in cellular level, we smartly synthesized TPP-fluorescein isothiocyanate (FITC) (shorten as TF) as a mitochondrial-targeting dye. As was shown in Figure S2 (B), TPP-COOH was reacted with FITC *via* simple ester reaction. The synthesized TF was characterized using ^1^H NMR (Figure S2 (A)) and MALDI-TOF MS (Figure S2 (C)), as well. The appearance of characteristic peak in ^1^H NMR (7.74-7.82 ppm, proton peak groups of benzoyl groups) initially indicated TF was synthesized successfully. The strongest intensity peak (*m*/*z* = 734.62530) was assigned as [M + H]^+^ of TF, suggesting TF was synthesized successfully.

*In vitro* stability assay was used to evaluate whether TD could stay stable in acidic tumor microenvironment. As shown in Figure S3, no obvious peak area changes for TD during the experiment period in pH 7.4, suggesting TD was stable in normal environment. Hopefully, TD also possessed excellent stability in pH 5.0 at 6 h, indicating TD could keep the structure in acidic tumor microenvironment at least 6 h and possess mitochondrial targetability into tumor cells. All these results supported favorable evidence for the efficient mitochondrial targetability of TD.

### 3.2. Preparation and characterization of liposomes

**Figure 1.**
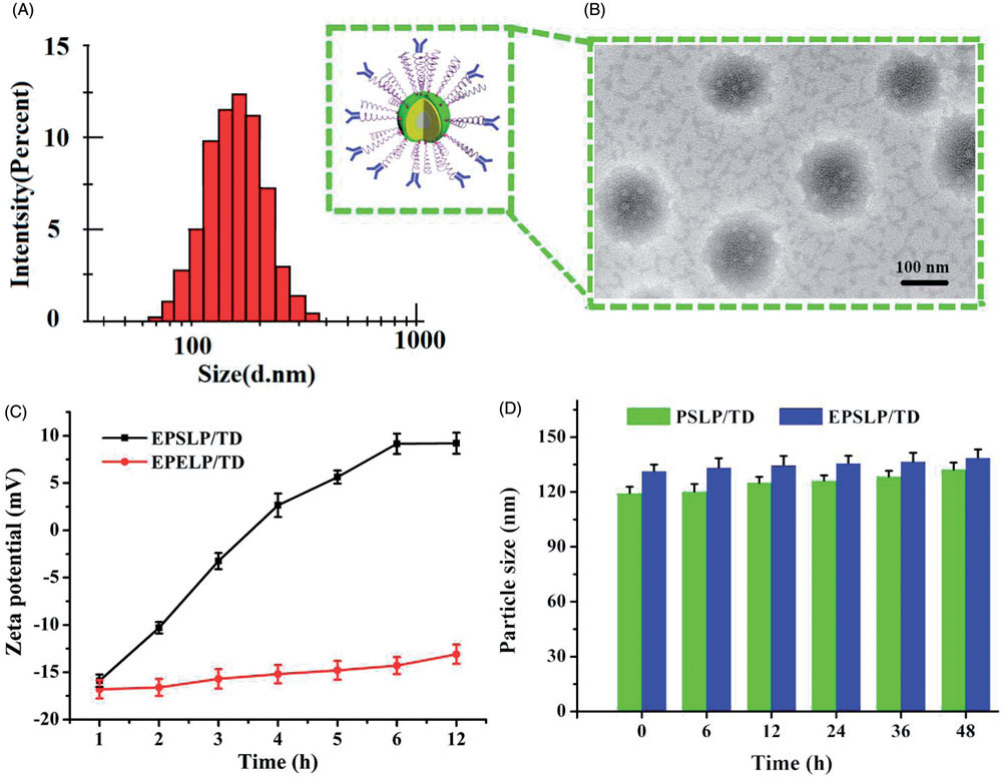
Physicochemical properties of liposomal formulations. (A) Particle size distribution of TD loaded liposomes (EPSLP/TD); (B) TEM images of EPSLP/TD; (C) zeta potential variation of EPSLP/TD and EPELP/TD at different pH values; (D) *In vitro* serum stability assay of liposomal formulations in the presence of 10% FBS at 37°.

Conventional thin-film hydration method was utilized to prepare different liposomes. Particle size, zeta potential, polydispersity index (PDI) and encapsulation efficiency (EE %) were tested and showed in Table 1. Particle size of blank liposomes (Blank Lip) was about 100 nm and zeta potential kept negative charge. When drugs were incorporated into liposomes, slight increase in particle size could be observed (∼110 nm). It is notable that zeta-potential of TD-loaded liposomes (LP/TD) verified from negative to positive, which increased to ∼9 mV. TPP group was exposed on the surface of the liposomes could be utilized to explain this phenomenon. It is well known that positive charge is a ‘double-edged-swords’ for nanocarrier. Because the negative charge of cell membrane, positive charge nanocarrier could increase cellular uptake and reach higher therapeutical effect. However, nonspecific toxicity would increase as well. In order to shield the positive charge and reduce its toxicity, PEGylated liposomes (PSLP/TD) were prepared. Hopefully, zeta-potential of PSLP/TD reduced to be neutral. Furthermore, zeta-potential of EphA 10 modified liposomes decreased significantly to approximately -16 mV. This strategy could not only reduce its nonspecific toxicity, but also increase its targetability. Particle size distribution of EPSLP/TD has been shown in Figure 1(A), the particle size of EPSLP/TD was about 120 nm with narrow size distribution. From the TEM images (Figure 1(B)), the liposomes showed a nearly spherical morphology with uniform particle size. The particle size of EPSLP/TD from TEM images was about 130 nm, which was in consistent with DLS data. In general, particle size of nanoparticles plays a vital role for further *in vivo* application. Narrow particle size distribution and small size of EPSLP/TD are very suitable for both *in vitro* and *in vivo* study.

**Table 1.** Characterization of different liposomes.

Formulations	Particle size (nm)	PDI	Zeta potential (mV)	EE %	LC %
Blank Lip	102.2 ± 1.6	0.213 ± 0.015	−10.26 ± 0.33	–	–
LP/DTX	108.2 ± 3.2	0.265 ± 0.018	−8.29 ± 0.21	69.11 ± 1.65	3.84 ± 0.09
LP/TD	110.2 ± 5.1	0.218 ± 0.062	9.12 ± 0.33	73.23 ± 3.54	4.07 ± 0.20
PSLP/DTX	115.3 ± 2.4	0.224 ± 0.011	−15.23 ± 0.31	68.52 ± 1.25	3.60 ± 0.07
PSLP/TD	118.6 ± 1.8	0.214 ± 0.021	−2.15 ± 0.14	72.15 ± 2.51	3.79 ± 0.13
EPSLP/DTX	129.3 ± 1.5	0.241 ± 0.033	−23.65 ± 0.54	69.24 ± 3.55	2.56 ± 1.25
EPSLP/TD	131.5 ± 2.6	0.252 ± 0.021	−16.25 ± 0.38	74.68 ± 1.68	3.83 ± 0.09
EPELP/TD	133.0 ± 1.5	0.102 ± 0.085	−17.10 ± 0.22	75.68 ± 1.14	3.88 ± 0.06

Our previous study demonstrated the novel cholesterol derivate PSC showed significant pH-sensitive characteristic (Chen et al., 2016). However, the mechanism of this phenomenon has not been extensively studied. Due to the positive charge of LP/TD (+9.12 mV) and negative charge of EPSLP/TD (-16.25 mV), we were interested to observe the changes of zeta-potential in different pH value. Mal-PEG_2000_-Cholesterol was used to replace PSC as negative control and EphA 10 was conjugated to Mal-PEG_2000_-Cholesterol to prepare pH-nonsensitive liposomes (EPELP/TD). As was illustrated in Figure 1(C), no significant zeta-potential changes could be observed in EPELP/TD group after incubation in PBS (pH 5.0) for 12 h. However, zeta-potential increased from −13 mV to 9 mV after incubation for EPSLP/TD. This interesting result could be explained by the cleavage of the pH-sensitive Schiff base bond. We can hypothesis that in acidic tumor microenvironment, Schiff base bond would hydrolyze and EphA 10-PEG outer shell would be removed from the surface of the liposomes, followed by the exposure of the positive charge. All these results indicated that EPSLP/TD showed pH-responsive capability *via* the cleavage of pH-sensitive bond.

The stability of liposomes is an important criterion for *in vivo* application. Unstable and easy to be eliminated in blood system is becoming the major barrier for clinical application of liposomes (Li et al., 2015c). Particle size verification of different formulations was measured in the presence of 10% FBS at 37 °C. As shown in Figure 1(D), both formulation groups (PSLP/TD and EPSLP/TD) showed a slight increase in particle size after incubation for 48 h, suggesting these liposomes were stable in blood system. The higher stability of both formulations were probably because PEG outer shell of EPSLP/TD or EPSLP/DTX would form a weak electrostatic repulsion and prevent the interaction between proteins and liposomes. Furthermore, this shell could also shield the positive charge and reduce binding capability between serum proteins and liposomal formulations.

### 3.3. *In vitro* release of liposomes

The extracellular pH (pH_e_) of most solid tumor ranged from 6.5 to 7.2, while normal blood remains to be pH 7.4. Furthermore, once liposomes were entered into cells, pH could drop as low as 5.5–6.0 in endosomes and 4.5–5.0 in lysosomes (Qiu et al., 2016b). Therefore, it is necessary to design pH-sensitive PEG derivate which could reach rapid release in tumor microenvironment. As was shown in Figure S4 (A), the release behavior of four different formulations was investigated. There was only approximately 40% drug released from the liposomes in normal pH (pH 7.4), while there was approximately 80% of drug released from liposomes in acidic pH (pH 5.0) for all formulations. According to previous study about the pH-sensitive mechanism of PSC, we can further demonstrate that the pH-sensitive of PSLP was because the hydrolysis of Schiff base bond and PEG shell was removed from the liposomes.

As was shown in Figure S4 (B), an interesting result could be found that the total release percentage of TD group was slightly lower than DTX group. This phenomenon was probably because TD possessed higher hydrophobicity, which showed higher hydrophobic strength between drug and phospholipid membrane. Taken these together, TD group showed lower release behavior.

Compared with PSLP groups, EPSLP groups showed lower release behavior. This was probably because EphA 10 layers were modified on the surface of the PEG shell and made the pH-sensitive Schiff base bond more difficult to contact with the environment. All these results demonstrated these novel drug delivery systems could stay relatively stable in normal system in order to reduce nonspecific toxicity while rapid release in acidic tumor microenvironment to reach higher therapeutical efficacy.

### 3.4. *In vitro* cytotoxicity

The cell viability of blank liposomes and different drug-loaded liposomal formulations were measured using standard MTT method, three different cell lines (MCF-7, A549 and HepG2) have been chosen to investigate its cytotoxicity. As was shown in Figure S5 (A), no significant cytotoxicity was found and the cell viability was all over 90% even in a high concentration (500 μg mL^−1^) among all cell lines. This result indicated that the cytotoxicity of different formulations was not attributed to the carriers themselves. Cell cytotoxicity of different liposomal formulations have been illustrated in Figure S5 (B), the cytotoxicity of different preparations showed dose-dependent characteristic against MCF-7 cell lines. IC_50_ of different groups have been shown in Figure S5 (C). Compared with DTX group, TD group showed higher cytotoxicity with lower IC_50_ value. This was probably because two major reasons: (1) positive charge of TD would increase the cellular uptake compared with DTX. (2) TPP functional group would target mitochondria to reach apoptosis-inducing effect. Compared with PSLP groups (PSLP/DTX and PSLP/TD), both EPSLP groups (EPSLP/DTX and EPSLP/TD) showed significantly higher cytotoxicity. It has been reported that there is a high expression of EphA 10 in breast tumors, but a low expression in most normal tissues (Zang et al., 2016). Therefore, the enhanced cell cytotoxicity was mainly attributed to the active-targetability of EphA 10.

To further confirm whether the enhanced cytotoxicity was resulted from EphA 10 mediated cell endocytosis, competitive cytotoxicity study was investigated through adding free EphA 10 antibody in culture medium with various concentration. As is illustrated in Figure S5 (D), compared with PSLP/DTX or PSLP/TD, there was a significant influence in EPSLP/DTX and EPSLP/TD with the concentration of EphA 10 increasing from 10^−3^ ∼ 10.0 μg mL^−1^. The cell cytotoxicity of EPSLP/DTX and EPSLP/TD decreased as the concentration of EphA 10 increased, indicating EphA 10 receptors of tumor cells were gradually occupied by free EphA 10. When the concentration of EphA 10 increased to 10.0 μg mL^−1^, there was no significant differences between PSLP/DTX and EPSLP/DTX, PSLP/TD and EPSLP/TD, respectively. These results further confirmed the enhanced cytotoxicity was ascribed to the EphA 10 receptor mediated cell endocytosis. Therefore, we could conclude that EphA 10 receptor plays an important role in targeted delivering antitumor drugs to tumor cells.

### 3.5. Cellular uptake study

Cellular uptake assay was used to evaluate targetability of different liposomal formulations using fluorescence microscopy and flow cytometry, qualitatively and quantitatively. FITC was utilized to replace DTX as a fluorescence probe. In addition, TF was firstly synthesized to replace TD to evaluate the mitochondria targetability of TPP functional groups. As was shown in Figure S6 (A), the image of both PSLP groups showed a relatively weak fluorescence intensity. In contract, intense green fluorescence intensity could be observed for both EPSLP groups. For further demonstrating whether the enhanced cellular uptake capability was attributed to EphA 10 modified on the liposomes, cells were preincubated with excess EphA 10 for 1 h. The images revealed that fluorescence intensity dramatically decreased to the same level as PSLP groups, suggesting that the higher cellular uptake was directly attributed to the EphA 10 receptor mediated cell endocytosis.

The amount of cellular uptake was also quantitatively analyzed by flow cytometry. As was shown in Figure S6 (B, C and D), cellular uptake of different liposomal formulations showed significant time-independent characteristic. EPSLP groups showed a significant increase in cellular uptake, which was 1.53- and 1.71-fold compared with PSLP group and EPSLP + EphA 10 group, respectively. These results demonstrated the higher amount cellular uptake was related to the EphA 10 receptor mediated cell endocytosis and these results were in consistent with MTT assay.

### 3.6. Apoptosis-inducing study

Cell apoptosis was considered to be a symbol of programed cell death. Therefore, we aimed to distinguish whether the cell death was due to apoptosis or necrosis. As was shown in Figure S7, control group showed minor apoptotic and necrotic cells, indicating the cells were in a good state. After incubation of DTX, TD, PSLP/TD and EPSLP/TD, apoptosis percentage increased to 24.4%, 52.9%, 67.5% and 82.7%, respectively. Compared with DTX, apoptosis cells could be significantly increased for TD group, suggesting that mitochondrial targeting moiety could efficiently induce cell apoptosis. In addition, compared with PSLP/TD, EPSLP/TD showed higher apoptosis percentage, which was mainly attributed to EphA 10-mediated cell endocytosis could increase the drug accumulation into cytoplasm and reach higher antitumor activity. All these results were in consistent with MTT assay,

### 3.7. Mitochondria targeting assay

**Figure 2.**
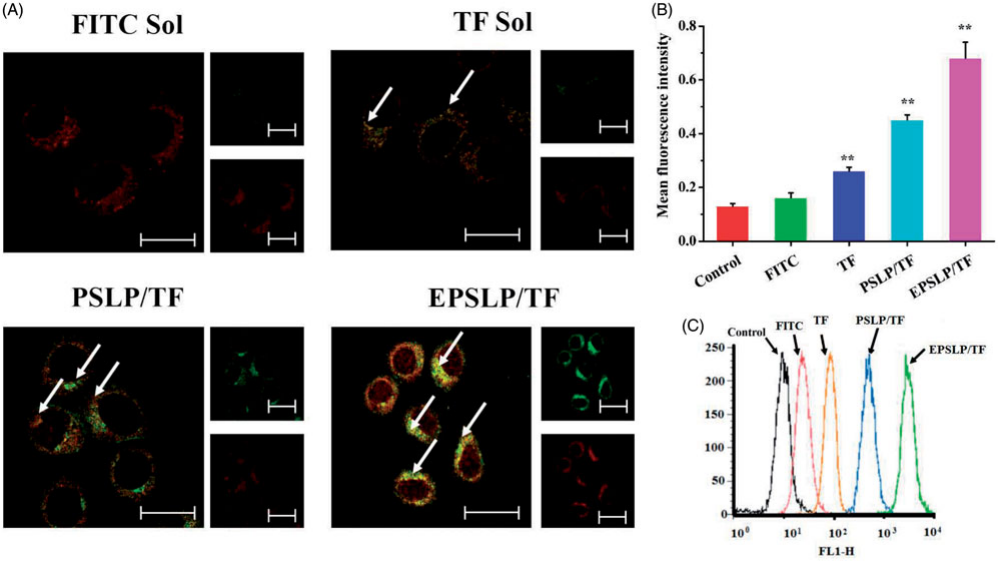
Mitochondrial targeting of liposomal formulations. (A) Mitochondrial co-localization of FITC (or TF) loaded liposomes in MCF-7 cell lines by confocal laser scanning microscopy. MCF-7 cells were incubated with different formulations (green pixel dots) and then stained the mitochondria with MitoTracker Red (red pixel dots). Yellow pixel dots in the merged pictures denote the colocalization of the probe within mitochondria compartments. (B and C) The accumulation of fluorescence probes into mitochondria against MCF-7 cells measured by flow cytometry. Data were presented as mean ± SD (*n* = 3), *p < .05, **p < .01. Scale bars represent 25 μm.

In order to evaluate the mitochondria targetability of novel conjugate TD and confirm the enhanced cytotoxicity was stemmed from mitochondria-medicated apoptosis pathway. Colocalization of mitochondria was examined with a confocal laser scanning microscope (CLSM). FITC and TF were used as a fluorescent dye to evaluate whether TPP functional group could accumulate into mitochondria. Mitotracker Red was used to stain mitochondria (red fluorescence) and green fluorescence was stemmed from the fluorescence dye. As was illustrated in Figure 2(A), after incubation for 2 h, fluorescence dye was dispersed into cytoplasm with green fluorescence, showing a co-localization with red fluorescence of mitochondria, which produced a yellow pixel dots in merge images. All TF group showed yellow fluorescence in different degree comparing with FITC group (no yellow fluorescence), indicating TPP moiety played an important role for efficient mitochondrial-targeting capability. PSLP/TF showed a stronger yellow fluorescence, indicating liposomes-based drug delivery system could enhance intracellular drug delivery efficacy. EPSLP/TF group showed the strongest yellow fluorescence intensity among all reference groups, suggesting that the higher amount of TF accumulated in mitochondria was attributed to EphA 10-mediated cell endocytosis and increased the drug accumulation in cytoplasm.

In order to quantitatively analyze the drug content from mitochondria, flow cytometry was used and mitochondria was separated from the cell using cell mitochondria isolation Kit. As was shown in Figure 2(B,C), the fluorescence intensity of different formulations were tested and there was a significant increase in TF group, PSLP/TF group and EPSLP/TF group compared with control group and FITC group. This result was in consistent with the CLSM assay. This interesting phenomenon could be explained by two distinct aspects. (1): EphA 10-mediated cell endocytosis could increase drug accumulation into cytoplasm. (2) TPP functional moiety could target mitochondria. This hierarchical targeted drug delivery system was expected to efficiently intracellular deliver antitumor drugs and facilitate its therapeutical effect.

Cell apoptosis is a process of programed cell death. The reduction of mitochondrial membrane potential (*△Ψm*) and the activation of caspases were considered to be a symbol of apoptosis (Youle and van der Bliek, 2012). Therefore, various apoptosis markers were measured after applying different formulations to confirm the cell death was due to apoptosis but not nurses or other reasons.

Mitochondrial membrane potential was measured using fluorescent JC-1 monomers. Green fluorescence could be observed when the dye was isolated into cytoplasm, while red fluorescence of aggregated JC-1 was observed when it was located in mitochondria depending on the mitochondrial membrane potential. Hence, the decrease of the relative intensity between green fluorescence and red fluorescence implied a decreased of *△Ψm.* As shown in Figure S8 (A and B), *△Ψm* were 0.65-, 0.42-, 0.27- and 0.14-fold lower than control group for DTX, TD, PSLP/TD and EPSLP/TD, respectively. TD showed higher influence in *△Ψm* comparing with DTX, indicating TD could efficiently target mitochondria and reduce mitochondria membrane potential. *△Ψm* for PSLP/TD and EPSLP/TD reduced significantly compared with both TD and DTX groups. This phenomenon suggested that liposomes could efficiently deliver drug to cytoplasm and increase drug accumulation into cytoplasm. Meanwhile, TD could target mitochondria and induce apoptosis effect, which showed a higher reduction of mitochondrial membrane potential. These results were in the same trend with pervious study.

Mitochondria-mediated cell apoptosis pathway is always triggered by various mechanisms. Among all of them, Bax/Bcl-2 channels polarizing, mPTP opening and mitochondrial membrane damage was considered to be the most favorable mechanism (Zunino and Storms, 2006; Dedkova and Blatter, 2012). When the cells were exposed to some antitumor drugs, the permeability of the mitochondrial membrane would increase, followed by a decrease in mitochondrial membrane potential. Subsequently, mitochondria would swell and cytochrome *c* was released from mitochondria. The released cytochrome *c* could activate the caspase 9, which in turn activate the downstream caspase 3 (Zhou et al., 2013). Caspase 3 could cleave poly (ADP-ribose) polymerase (PARP), which was a major biomarker for apoptosis, and lead to cell death (Hocsak et al., 2017; Mishra and Kowluru, 2017). Therefore, a series of protein expressions were investigated to evaluate the influence of different formulations. As was shown in Figure S9 (A), protein expressions of pro-apoptosis protein Bax and anti-apoptosis protein Bcl-2 were investigated, firstly. Compared with negative control, expression of Bax protein increased in different degree for different drug-treated groups and Bcl-2 expression of different groups decreased, as well. These results demonstrated that cell apoptosis was depended on the mitochondria-mediated cell apoptosis pathway. In addition, Cas-3 and Cas-9 expression were investigated, EPSLP/TD group showed the highest Cas-3 and Cas-9 expression compared with other groups, reflecting EPSLP/TD could efficiently accumulate in cytoplasm *via* EphA 10 mediated cell endocytosis and induce cell apoptosis *via* mitochondria-mediated apoptosis pathway. PARP and cleaved-PARP (C-PARP) were investigated as well. EPSLP/TD showed the lowest PARP expression and the highest C-PARP expression. This phenomenon was probably because the highest Cas-3 expression for EPSLP/TD, which would cleave the PARP to C-PARP. Release of cytochrome *c* was also measured using immunofluorescence. As was shown in Figure S9 (B), blue fluorescence represents nucleus, while red fluorescence represents released cytochrome *c* in tumor cells. There was almost no red fluorescence in control group, suggesting that the cell was in good condition. Different drug-treated groups showed different cytochrome *c* expression and EPSLP/TD showed the highest expression. All these results were in consistent with the mitochondrial membrane potential (*△Ψm*) assay and further demonstrated EPSLP/TD could efficiently induce cell apoptosis *via* mitochondria-mediated apoptosis pathway.

### 3.8. *In vivo* biodistribution assay

From the results above, we could find TD possessed excellent cytotoxicity and apoptosis inducing effect against MCF-7 cell line. However, there are many barriers for efficient drug delivery to tumor sites *in vivo* such as easy to be eliminated by RES, easy to interact with blood proteins and nonspecific toxicity. Therefore, PEGylated liposomes have been extensively used in drug delivery system. In the *in vivo* assay, we evaluated the tumor targetability of different formulations using real-time fluorescence IVIS imaging system in nude mice-bearing MCF-7 tumor cells. DiR were used to replace DTX or TD to evaluate the *in vivo* biodistribution behavior of liposomes-based nanocarriers. Mice were injected with DiR solution, PSLP/DiR or EPSLP/DiR intravenously through tail vein with the same dosage of DiR reagent. As was shown in Figure S10, DiR solution group showed a whole body biodistribution after administration for 6 h. Thereafter, the signal decreased dramatically and the signal could be observed in liver and kindly, which had abundant blood flow, after postinjection for 48 h. In comparison, PSLP/DiR group showed a relative stronger fluorescence intensity in tumor and liver, suggesting PEGylated liposomes could enhance circulation time. Hopefully, EPSPLP/DiR group possessed excellent targetability to tumor site. There was a strong fluorescence intensity in tumor sites after administration for 6 h, the signal gradually increased in 12 h in tumor sites and sustained for up to 48 h, reflecting the increased targetability in EPSLP/DiR. The stronger targetability of EPSLP/DiR was probably because the two reasons. (1) PEGylated liposomes could decrease the elimination by RES thus enhance circulation time. (2) EphA 10 mediated cell endocytosis could enhance cell reorganization for EPSLP/DiR and increase cellular uptake. These results were in consistent with the *in vitro* cellular uptake assay and demonstrated EPSLP could reach active targetability *in vivo* as well.

### 3.9. *In vivo* antitumor activity

**Figure 3.**
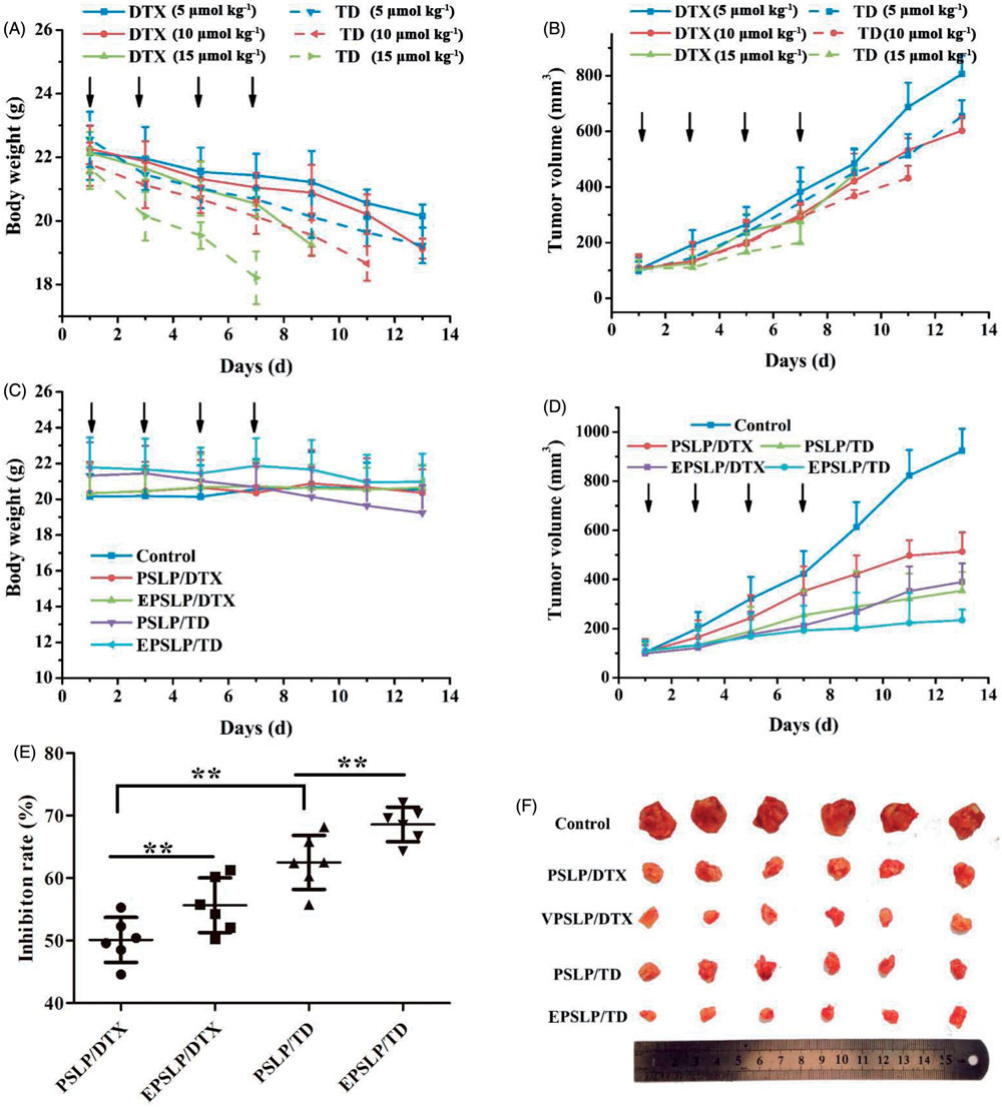
*In vivo* antitumor efficacy of different formulations against MCF-7 tumor-bearing mice (*n* = 6). Tumor volume changes (A) and body weight variation (B) of the tumor-bearing mice with different DTX and TD dosage. Body weight (C) and tumor volume changes (D) of the mice when they were intravenously injected PSLP/DTX, PSLP/TD, EPSLP/DTX and EPSLP/TD with the TD or DTX concentration was 5 μmol kg^-1^. At the end of the trial, tumor tissues were isolated and inhibition rate (E) was calculated. Meanwhile, tumor images (F) of different liposomal formulations were photographed. Note: *p < .05, **p < .01.

*In vivo* antitumor assay was performed to evaluate the antitumor activities of different formulations. As was shown in Figure 3(A,B), the dose of DTX or TD administration was optimized, firstly. Enhanced antitumor activities were observed as the dose increased, demonstrating the antitumor activities possessed dose-independent feature. Compared with DTX group, tumor size of TD group decreased significantly, which were 1.23-, 1.39- and 2.23-fold lower than DTX (5 μmol kg^−1^) DTX (10 μmol kg^−1^) and DTX (15 μmol kg^−1^), respectively. These results demonstrated TD owned favorable *in vivo* antitumor activities. Unfortunately, body weight of all groups decreased dramatically. All mice were dead on the 9th day for DTX (15 μmol kg^−1^), 11th day for TD (10 μmol kg^−1^) and 7th day for TD (15 μmol kg^−1^), respectively. These results reflected both DTX and TD owed severely systematic toxicity. Furthermore, TD group showed higher systematic toxicity compared with DTX groups, this phenomenon could be explained by the positive charge functional group TPP conjugated with DTX, which lead to the reorganization of RES and increased elimination rate, followed by higher systematic toxicity. Therefore, the optimized dose administration was 5 μmol kg^−1^ and the optimal dose administration was applied for evaluation the antitumor activities of different drug-loaded liposomes.

As was shown in Figure 3(C,D), the growth of all liposomes groups was lower than control group. Compared with PSLP/DTX group, EPSLP/DTX could significantly inhibit tumor growth. Compared with both DTX groups, both TD groups showed higher tumor inhibition efficacy. There was no obvious decrease in body weight for all the groups due to its pH-responsive drug release, followed by reducing severe side effect. The tumor growth rate of all groups has been shown in Figure 3(E,F), EPSLP/TD group showed a lower growth rate (26.14%) compared with PSLP/TD (36.53%), EPSLP/DTX (40.23%) and PSLP/DTX (53.64%). Excellent antitumor activity of EPSLP/TD group was probably because EphA 10 modified on the surface of liposomes could enhance targetability to solid tumor and higher amount of drug could accumulate in tumor sites. Furthermore, TD could specifically target mitochondria of tumor cells and induce apoptosis effect.

### 3.10. Histology, immunofluorescence and immunohistochemistry assay

**Figure 4.**
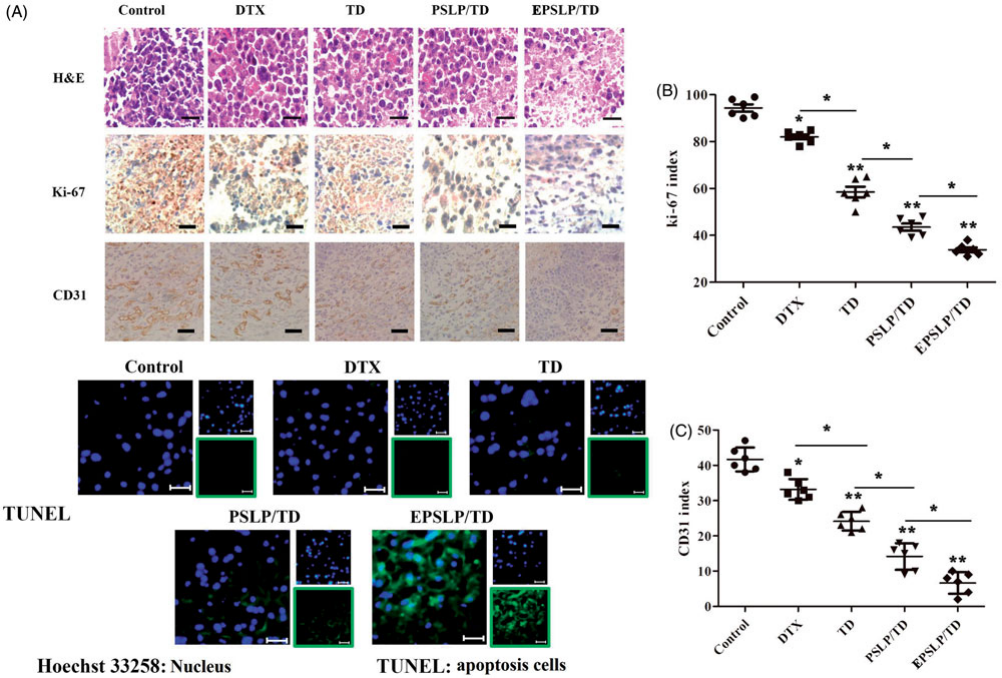
Tumors from previous study were isolated, fixed using formalin and prepared paraffin sections for histological study. H and E staining, Ki-67 immunohistochemistry (IHC), CD31 IHC and TUNEL immunofluorescence of tumor tissues were carried out to evaluate the antiproliferation, anti-angiogenic and apoptosis-inducing effect of different formulations (A). Quantification of proliferative cells from six random fields (brown and blue pixel dots represent the proliferative cells and the nucleic, respectively). The Ki-67 index was calculated as the ratio of proliferative cells to total cells in each field (B). Quantification of angiogenic cells from six random fields (brown and blue pixel dots represent the tumor vessel and the nucleic, respectively). The CD31 index was calculated as the ratio of angiogenic cells to total cells in each field (C). *p < .05, **p <.01. Scale bars represent 100 μm for CD31 IHC and 50 μm for other groups.

To further demonstrate the antitumor activity of different formulations in histological level, various markers have been investigated. As was shown in Figure 4(A), H and E staining assay confirmed the apoptosis and necrosis areas were the greatest for EPSLP/TD (with nucleic shrinkage and presence of voids in tumor section). These results were in consistent with the *in vivo* antitumor activities above.

It has been reported that Ki-67 is a nuclear protein with nuclear function, which is expressed in all phases of the cell cycle except G_0_ phase. Therefore, Ki-67 has become the major markers of tumor proliferation (Ayed et al., 2014; Yamazaki et al., 2015). In this study, Ki-67 immunohistochemistry (Ki-67 IHC) was used to evaluate the proliferation level of different drug treated groups. As was shown in Figure 4(A), brown pixel indicated the expression of Ki-67, while blue pixel dots represented the nucleic. Tumor images indicated that there was only a slight decrease in expression of Ki-67 for DTX and TD groups compared with control group. However, PSLP/TD and EPSLP/TD groups showed a significant decrease in the expression of Ki-67. This interesting result demonstrated liposomes could efficiently deliver drugs to tumor sites and possess anti-proliferation effect. Furthermore, EPSLP/TD showed a higher anti-proliferation effect compared with PSLP/TD, demonstrating EphA 10 modified on the surface of the carrier showed an excellent active targetability to tumor tissues. The Ki-67 index was also quantitatively analyzed as Figure 4(B). EPSLP/TD showed the lowest Ki-67 index (34.23 ± 2.59%) compared with other groups and this result was in correlation with images.

Tumor increase requires an ample supply of oxygen and nutrients from blood vessels to support their proliferation. Higher proliferation rate of tumor would also stimulate angiogenesis. It has been reported that the enhanced delivery of docetaxel could efficiently reduce angiogenesis and metastasis respectively (Kutty et al., 2015). Therefore, we were interested to observe whether TD could possess anti-angiogenesis properties. We investigated the anti-angiogenic effect of different formulations on tumor sites with CD31 immunohistochemistry (IHC), a blood vessel marker. As shown in Figure 4(A), brown pixel indicated the blood vessels of tumor sites. Compared with control group, CD31 staining of all drug treated groups were notably lower. Intense staining of CD31 was observed for tumor treated with DTX and TD. This result was mainly because insufficient delivery of drugs, resulting in a weak anti-angiogenic effect. In comparison, both formulation groups showed significant anti-angiogenic effect, demonstrating TD could also efficient reduce angiogenic effect. Furthermore, EPSLP/TD showed a higher anti-angiogenic effect compared with PSLP/TD group, suggesting EPSLP/TD could increase cellular uptake *via* receptor-mediated cell endocytosis and reach higher accumulation in tumor tissues. Figure 4(C) illustrated the CD31 index of different groups, EPSLP/TD showed the lowest CD31 index (8.79 ± 3.07%) among all reference groups. These findings indicated that TD could efficiently reduce angiogenic effect and EPSLP could deliver TD to tumor tissue with a higher accumulation concentration and reach higher therapeutical effect.

Apoptosis is a cell death process with specific distinct morphologic, biochemical, and molecular alterations. We have examined the apoptosis-inducing effect of different formulations *via* PI/Annexin V-FITC method in cellular level. However, these results could not entirely replace *in vivo* studies. Therefore, TUNEL assay was used to confirm the apoptosis effect of different drug-treated groups. As was shown in Figure 4(A), green fluorescence signal of free DTX and TD groups increased slightly compared with control groups, demonstrating free DTX and TD had a slight apoptosis inducing effect *in vivo*. However, green signal increased dramatically for PSLP/TD and EPSLP/TD, suggesting the enhanced delivery of drug could significantly induce apoptosis of tumor tissue. These results were in consistent with the *in vitro* apoptosis study.

### 3.11. *In vivo* toxicity assay

The major problem of chemotherapeutics is the systematic toxicity, higher dose of antitumor drugs would cause damage to normal tissues. Therefore the *in vivo* toxicity assay was investigated. Healthy BALB/c mice were injected with different formulations (5 μmol kg^−1^ of DTX or TD) through tail vein once every 2 days. Clinical signs of toxicity and weight loss were recorded. After treatment, main organs were harvested and stained using H and E. There was no significant difference in body weight among all the groups and the body weight increased slightly during the trial (Figure S11 (A)). Compared with negative control, no significantly signs of dehydration, locomotors impairment, muscle loss, anorexia and other symptoms associated with animal toxicity related to abnormal behavior occurred. Meanwhile, no obvious histological verification associated with systematic toxicity could be observed compared with control groups (Figure S11 (B)). These results could make sure this dose of drug was safety after administration through tail vein.

## 4. Conclusions

In this paper, novel mitochondria-targeted derivate TD has been firstly synthesized and incorporated it into the liposomes which possessed significant pH-sensitive characteristic and active targetability against MCF-7 cell line. Increased cytotoxicity of EPSLP/TD could be observed in MCF-7 cells due to efficient delivery antitumor drug to mitochondria of tumor cells and induce mitochondria-mediated apoptosis pathway. In order to evaluate the mechanism of apoptosis inducing effect, a series trials were carried out and results indicated EPSLP/TD could accumulate into MCF-7 cells *via* receptor-mediate endocytosis and efficient deliver TD to mitochondria, which possessed hierarchical targetability. In addition, EPSLP/TD could decrease mitochondrial membrane potential, increase cytochrome *c* release to cytosol and activate Cas-9/3, finally induce cell apoptosis. Meanwhile, a series of *in vivo* assays were also carried out and the results suggested EPSLP/TD could specifically accumulate in tumor region, which showed excellent antitumor activity *in vivo*. Furthermore, this preparation possessed excellent anti-angiogenesis effect, antiproliferation effect and apoptosis effect *in vivo*. Therefore, EPSLP/TD is a promising antitumor drug delivery system which possessed hierarchical targetability for treatment of breast cancer both *in vitro* and *in vivo*.

**Scheme 1.**
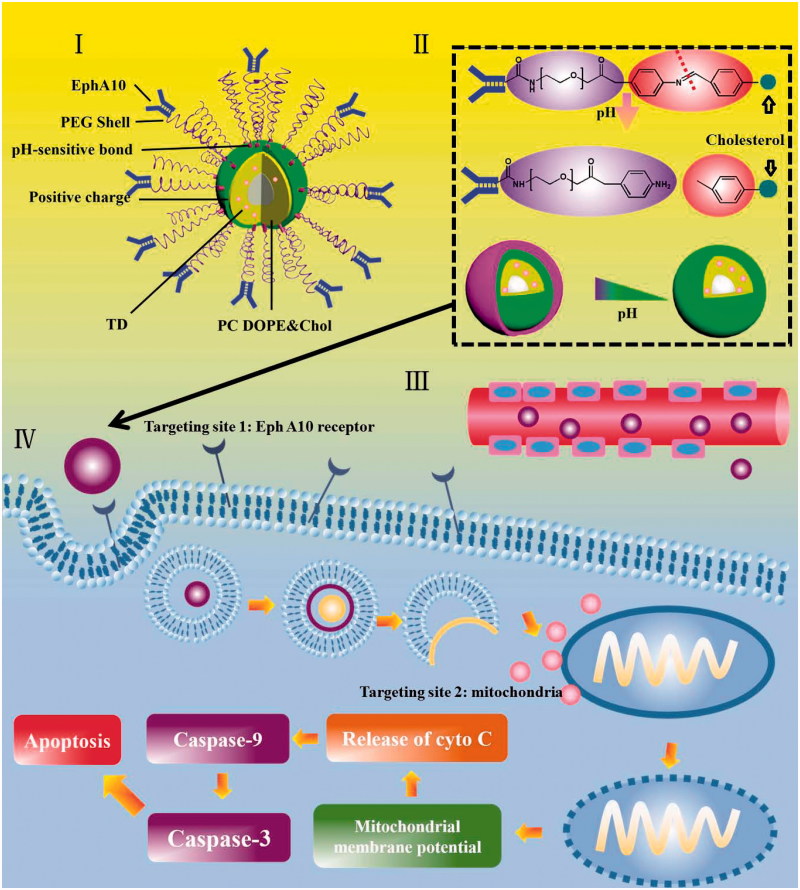
Schematic illustration of multifunctional liposomes (EPSLP/TD) with hierarchical tumor targetability and pH-responsive capability to MCF-7 cell line. (I) Composition illustration of EPSLP/TD. (II) pH-responsive mechanism of EPSLP/TD in acidic tumor microenvironment. (III) EPR effect of EPSLP/TD after intravenous administration. (IV) EPSLP/TD was internalized into MCF-7 tumor cells *via* EphA10 receptor-mediated endocytosis, followed by endosome escape and target mitochondria, which induced the decrease of mitochondrial membrane potential, released of cytochrome *c*, activated Cas-9 and Cas-3, finally inducing cell apoptosis.

## Supplementary Material

IDRD_Chen_et_al_Supplemental_Content.docx
