# Supplementary material for: Eph A10-modified pH-sensitive liposomes loaded with novel triphenylphosphine–docetaxel conjugate possess hierarchical targetability and sufficient antitumor effect both in vitro and in vivo
Source: Drug Deliv. 2018 Mar 7;25(1):723–37. doi: 10.1080/10717544.2018.1446475 (PMC6058733; doi:10.1080/10717544.2018.1446475)
Supplement: IDRD_Chen_et_al_Supplemental_Content.docx [file IDRD_A_1446475_SM4959.docx]

*Supplementary Data*

**Eph A10 modified pH-sensitive liposomes loaded with novel triphenylphosphine-docetaxel conjugate possess hierarchical targetability and sufficient antitumor effect both *in vitro* and *in vivo***

Jiulong Zhang^a^, Chunrong Yang^b^, Shuang Pan^a^, Menghao Shi^a^, Jie Li^c^, Haiyang Hu^a^, Mingxi Qiao^a^, , Dawei Chen^a*^and Xiuli Zhao^a*^.

^a^ School of Pharmacy, Shenyang Pharmaceutical University, 103 Wenhua Road, Shenyang, Liaoning, PR China, 110016

^b^ College Pharmacy of Jiamusi University, Xuefu Street No.148, Jiamusi, Heilongjiang, PR China, 154007

^c^ Mudanjiang Medical University, Tongxiang Street No.3, Mudanjiang, Heilongjiang, PR China, 157011

**E-mail**: [zjl1160@163.com](mailto:zjl1160@163.com) (J. Zhang); [raura3687yd@163.com](mailto:raura3687yd@163.com) (X. Zhao)

**Note**: Xiuli Zhao* and Dawei Chen* are both corresponding authors.

**Keywords**: pH-responsive; Docetaxel conjugate; Multifunctional liposomes; Hierarchical targetability; Anticancer efficacy


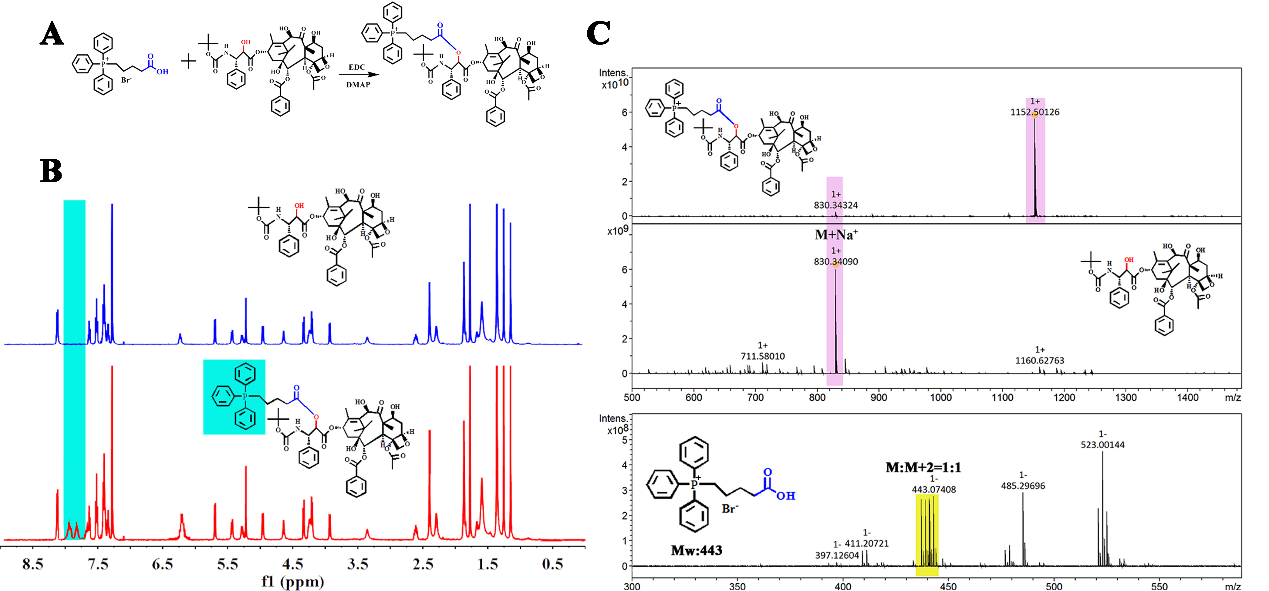


**Fig.S1**: Characterization of novel triphenylphosphine-docetaxel (TD) conjugate. (A) Synthesis pathway of TD; (B) Typical ^1^H-NMR spectrum of DTX and TD, respectively; (C) Matrix assisted laser desorption ionization-time of flight mass spectrum (MALDI-TOF-MS) of TD, DTX and TPP-COOH, respectively.


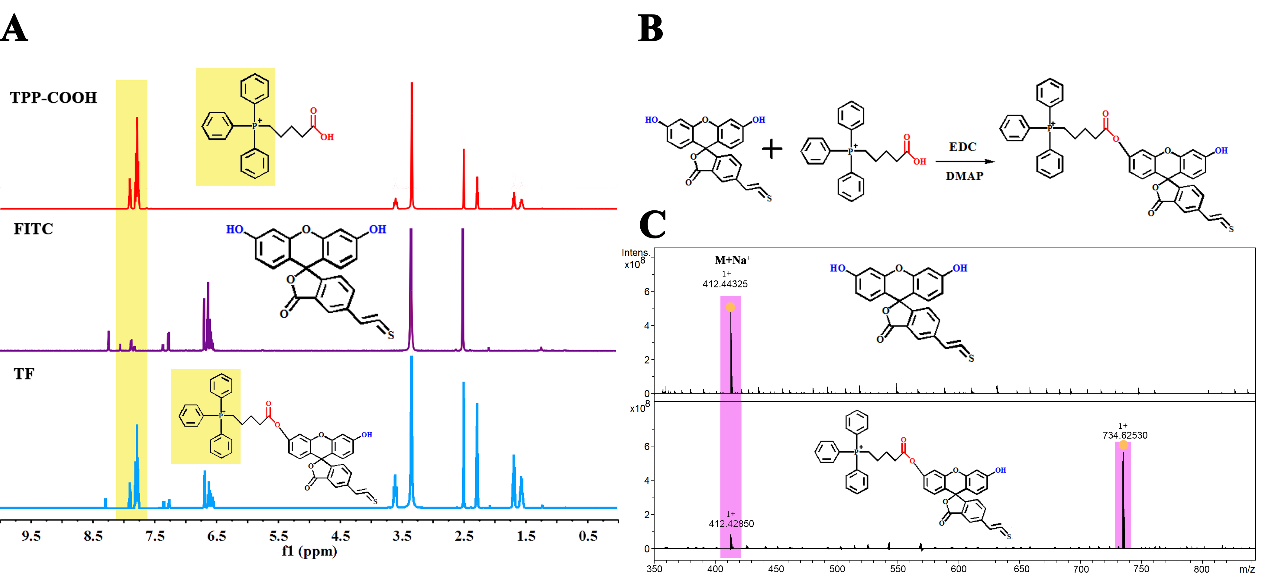


**Fig.S2**: Characterization of triphenylphosphine- fluorescein isothiocyanate (TPP-FITC, shorten as TF). (A) Typical Typical ^1^H-NMR spectrum of TPP-COOH, FITC and TF, respectively. (B) Synthesis pathway of TF. (C) MALDI-TOF-MS of FITC and TF.


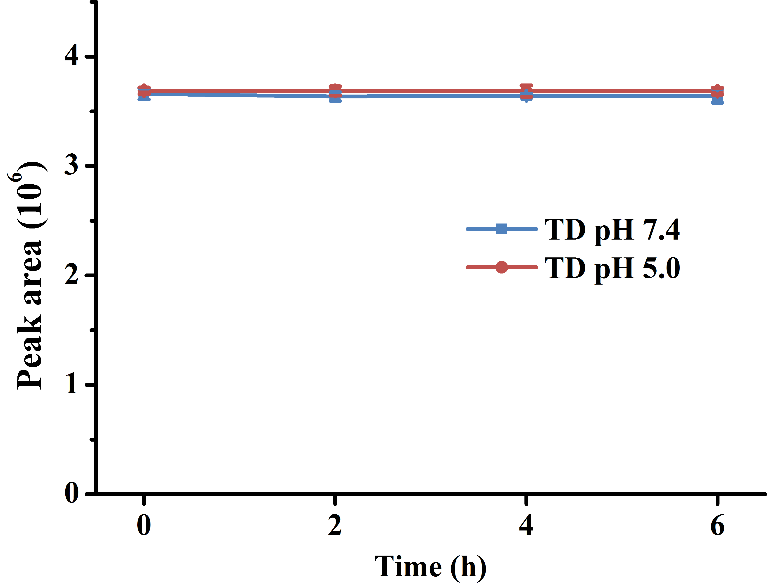


**Fig.S3**: *In vitro* stability of TD in different pH value (pH 7.4 and 5.0) using HPLC method


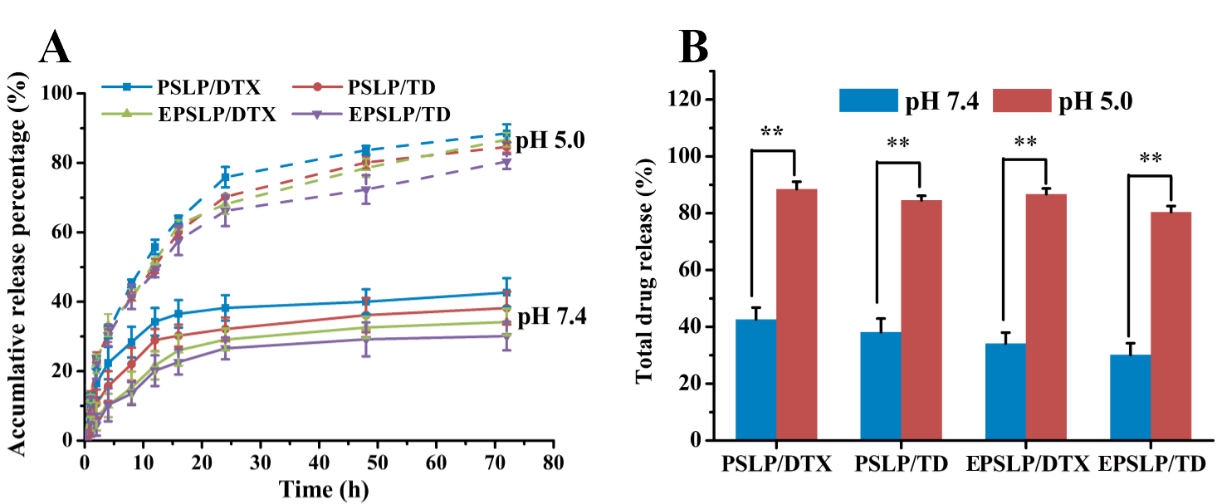


**Fig.S4**: *In vitro* drug release of different liposomal formulations in different pH at 37℃(A) and the total drug release profiles of different formulations (B) at different pH at 37℃.(mean±SD, n=3)


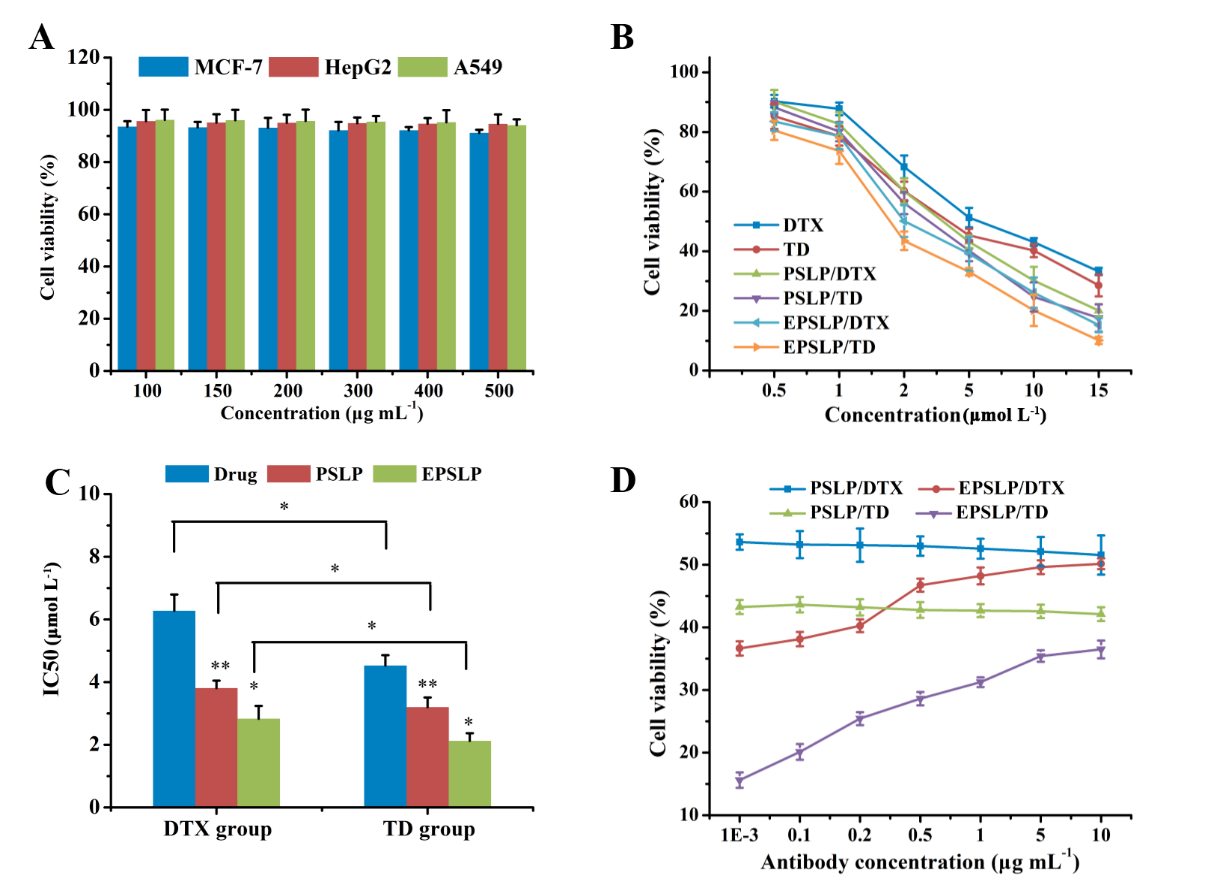


**Fig.S5**: Cell cytotoxicity of different formulations using standard MTT assay against different cell lines.(A) *In vitro* cytotoxicity of blank liposomal formulations against different cell lines; (B) *In vitro* cytotoxicity of different formulations against MCF-7 cells; (C) IC_50_ value of different formulations against MCF-7 cell lines; (D) Competitive cytotoxicity of different formulations against MCF-7 cell line with an increase of antibody concentrations.(n=3)


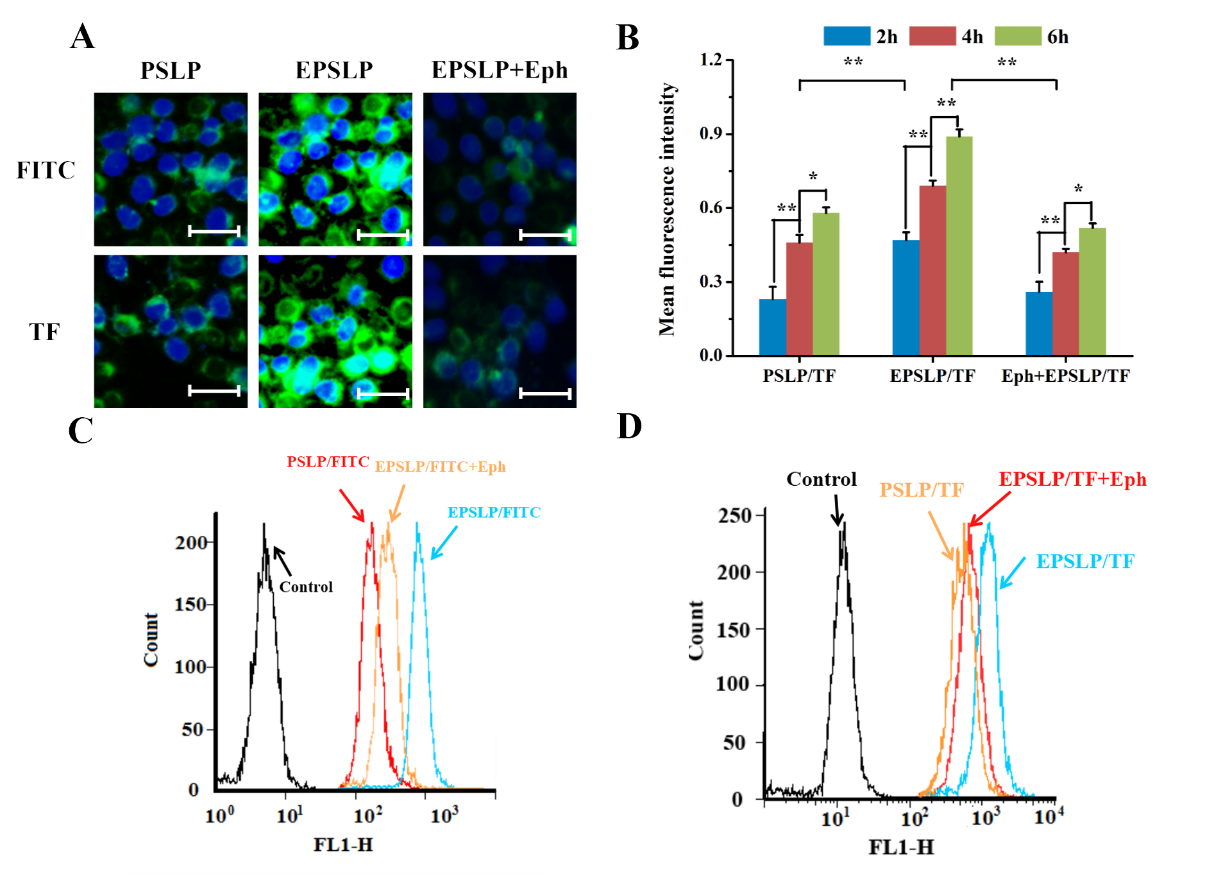


**Fig.S6**: Cellular uptake assay of different fluorescence probe-loaded liposomal formulations against MCF-7 cells. (A) Fluorescence microscopy images of different FITC or TF loaded liposomal formulations in the presence or in the presence of Eph A10 antibody. Green and blue fluorescence indicated FITC (or TF) and Hoechst 33258, respectively. Scale bars represent 50 μm. Flow cytometry analysis of cellular uptake in different time point in the presence or in the absence of EphA 10 against MCF-7 cell lines (B) (C) and (D). **p*<0.05, ***p*<0.01.


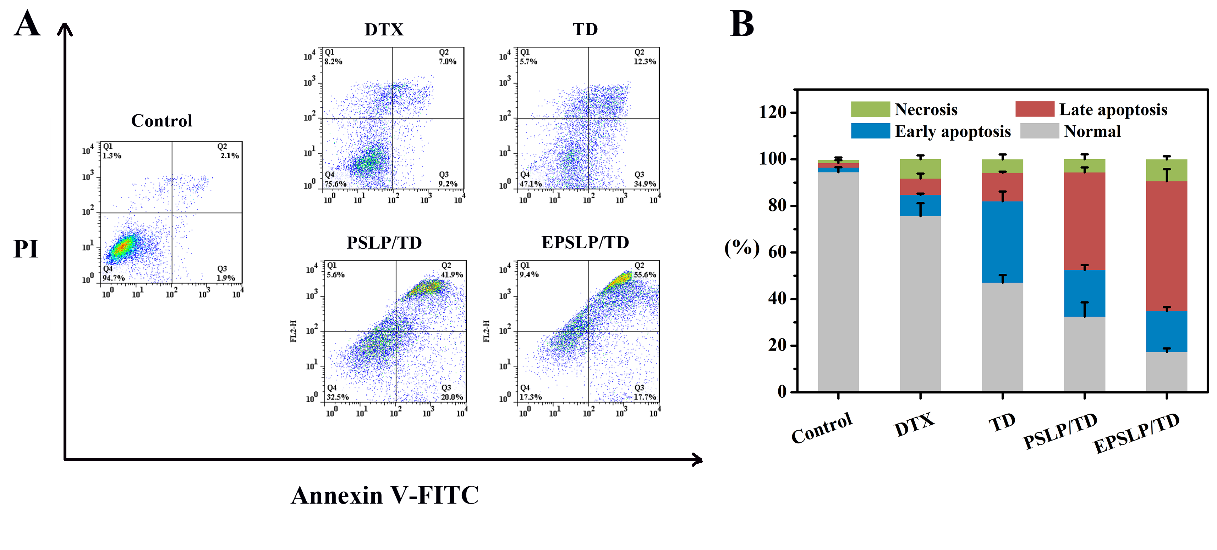


**Fig.S7**: Apoptosis inducing effect of different formulations against MCF-7 cells. (A) Apoptosis profiles of MCF-7 cells after incubation with control, DTX, TD, PSLP/TD and EPSLP/TD, respectively. (B) Quantitatively analyze the number of cells in different quadrants of different groups (n=3).


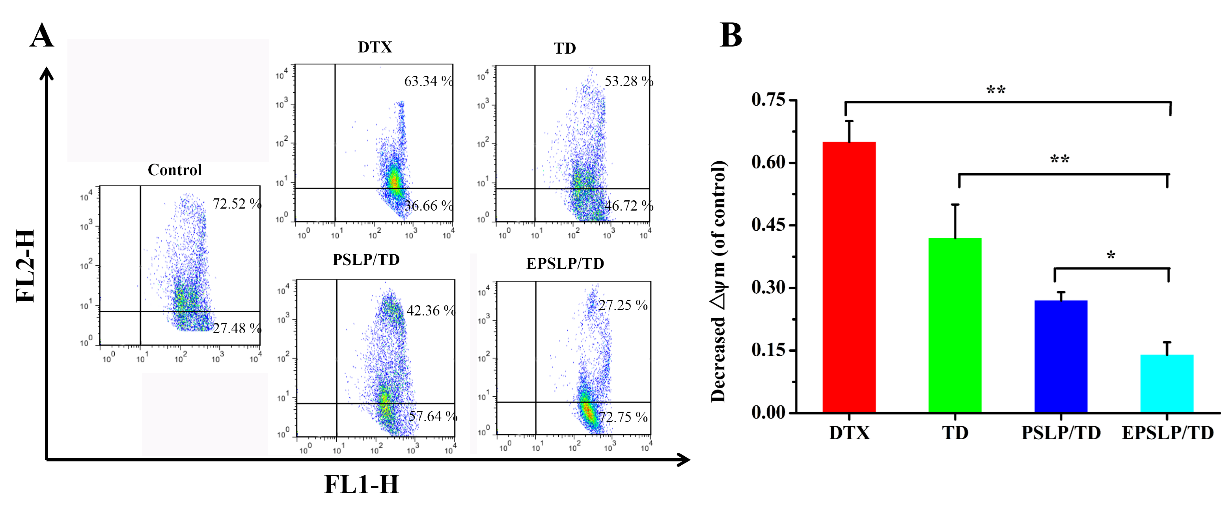


**Fig.S8**: Mitochondria-dependent apoptosis pathways. (A) Relative fluorescence intensity of JC-1 aggregates and monomer measured by flow cytometry after applying control, DTX, TD, PSLP/TD and EPSLP/TD, respectively. (B) The change in mitochondria membrane potential (*△Ψ*m) of MCF-7 cells after applying DTX, TD, PSLP/TD and EPSLP/TD compared with control group. Data were presented as mean±SD (n =3). **p* < 0.05, ***p* < 0.01.


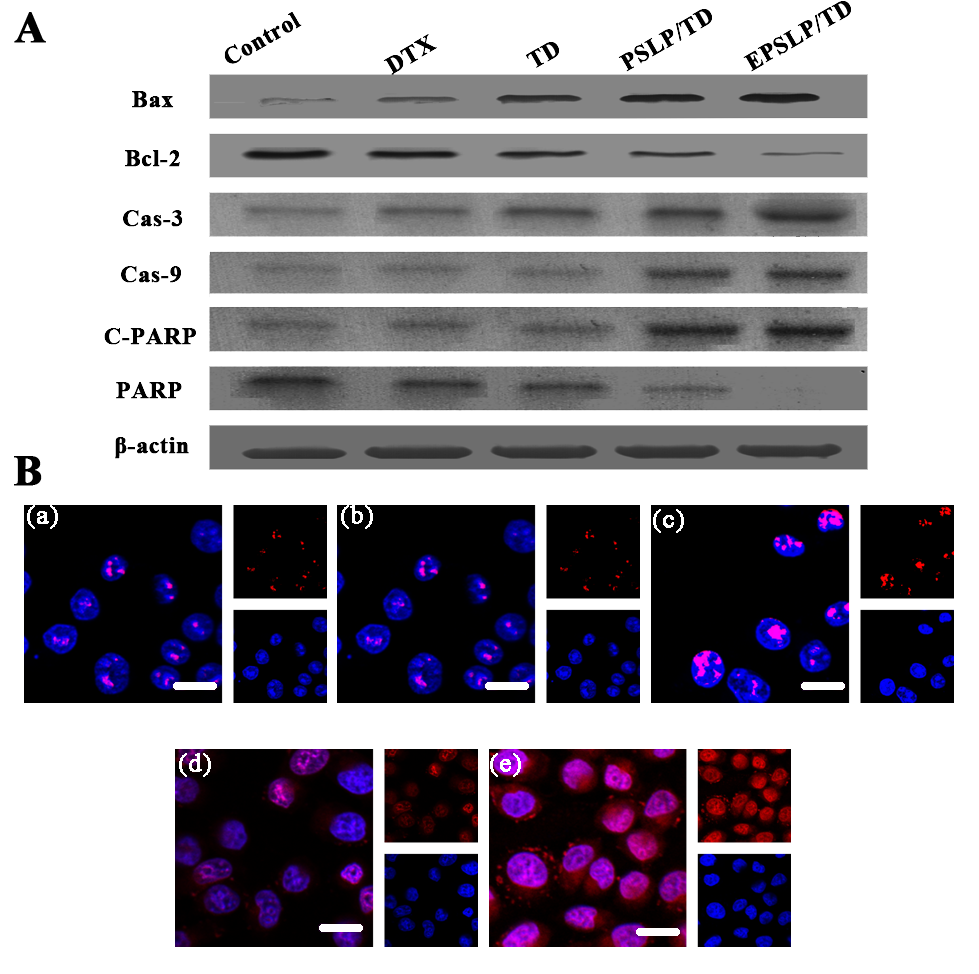


**Fig.S9**: Mitochondria-dependent apoptosis pathways. (A) The influence of apoptosis relative protein (Bax, Bcl-2, Cas-3, Cas-9, cleaved PARP, PARP) against MCF-7 cells after applying with various formulations measured by Western immunoblotting method. (B) Immunofluorescence staining images of cytochrome *c* translocated from mitochondria to cytosol after applying with culture medium (a), DTX (b), TD (c), PSLP/TD (d) and EPSLP/TD (e), respectively. Scale bars represent 50 μm. Green and blue fluorescence indicated the released cytochrome *c* and nucleic, respectively.


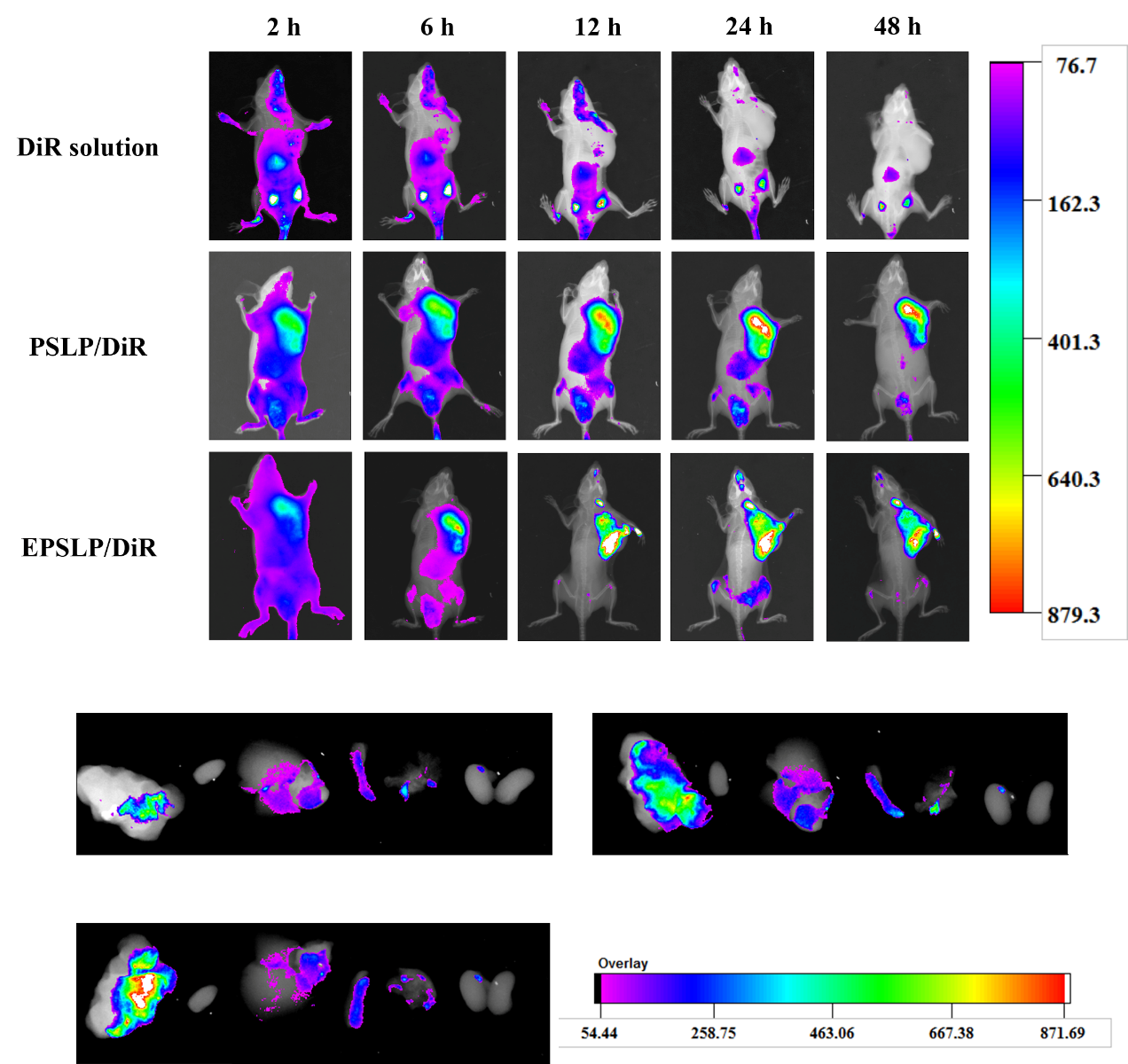


**Fig.S10**: Real-time imaging of liposomal formulations (EPSLP) facilitated the uptake of the preparations into MCF-7 tumor-bearing SCID mice. The mice were intravenously injected different DiR-loaded liposomes with the same DiR concentration. Major organs were obtained and observed at the end of the study.


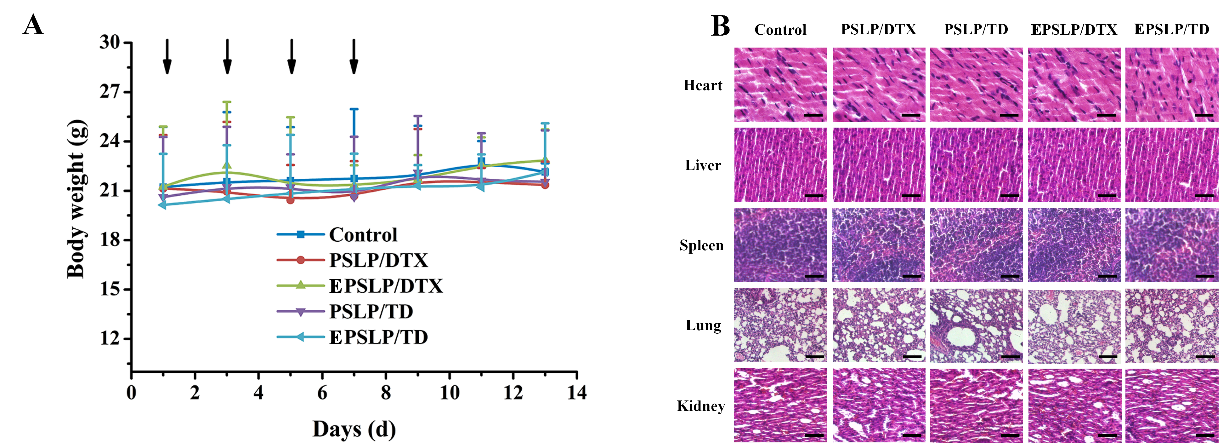


**Fig.S11**: *In vivo* toxicity evaluation of different liposomal formulations against normal mice. (A) Body weight curves of different groups (concentration of DTX or TD was 5 μmol kg^-1^). (B) H&E staining of main organs of normal mice with different treatments. Scale bars represent 50 μm.
